# Label-Free Protein-RNA Interactome Analysis Identifies Khsrp Signaling Downstream of the p38/Mk2 Kinase Complex as a Critical Modulator of Cell Cycle Progression

**DOI:** 10.1371/journal.pone.0125745

**Published:** 2015-05-20

**Authors:** Jorge Boucas, Christian Fritz, Anna Schmitt, Arina Riabinska, Lisa Thelen, Martin Peifer, Uschi Leeser, Peter Nuernberg, Janine Altmueller, Matthias Gaestel, Christoph Dieterich, H. Christian Reinhardt

**Affiliations:** 1 Department I of Internal Medicine, University Hospital of Cologne, Weyertal 115B, 50931, Cologne, Germany; 2 Cologne Excellence Cluster on Cellular Stress Response in Aging-Associated Diseases, University of Cologne, Weyertal 115B, 50931, Cologne, Germany; 3 Center for Molecular Medicine Cologne (CMMC), University of Cologne, Cologne, Germany; 4 Department of Translational Genomics, University of Cologne, Cologne, Germany; 5 Cologne Center for Genomics (CCG), University of Cologne, Cologne, Germany; 6 Institute of Biochemistry, Hannover Medical School, Hannover, Germany; 7 Computational RNA Biology and Ageing, Max Planck Institute for Biology of Ageing, Joseph-Stelzmann Straße 9b, 50913, Cologne, Germany; The University of Hong Kong, HONG KONG

## Abstract

Growing evidence suggests a key role for RNA binding proteins (RBPs) in genome stability programs. Additionally, recent developments in RNA sequencing technologies, as well as mass-spectrometry techniques, have greatly expanded our knowledge on protein-RNA interactions. We here use full transcriptome sequencing and label-free LC/MS/MS to identify global changes in protein-RNA interactions in response to etoposide-induced genotoxic stress. We show that RBPs have distinct binding patterns in response to genotoxic stress and that inactivation of the RBP regulator module, p38/MK2, can affect the entire spectrum of protein-RNA interactions that take place in response to stress. In addition to validating the role of known RBPs like Srsf1, Srsf2, Elavl1 in the genotoxic stress response, we add a new collection of RBPs to the DNA damage response. We identify Khsrp as a highly regulated RBP in response to genotoxic stress and further validate its role as a driver of the G_1_/S transition through the suppression of *Cdkn1a^P21^* transcripts. Finally, we identify KHSRP as an indicator of overall survival, as well as disease free survival in glioblastoma multiforme.

## Introduction

In response to genotoxic stress, cells activate a complex, kinase-based signaling network, which is commonly referred to as the DNA damage response (DDR) [1, 2]. To ensure that DNA has been fully replicated in an undamaged state before distribution to both daughters, cells progress through a series of highly conserved cell cycle checkpoints prior to mitosis [3–5]. These checkpoints allow time to repair genotoxic lesions, or, if damage is excessive, lead to the induction of apoptosis [3, 4]. Thus, checkpoint signaling can be seen as an effective fail-safe mechanism to provide genome stability through cell cycle arrest with subsequent DNA repair, or apoptotic elimination of mutated, incipient cancer cells. The canonical DDR network consists of two major kinase signaling branches, which operate through the upstream kinases ATR (ATM-Rad3 related) and ATM (ataxia telangiectasia mutated), as well as their downstream effector kinases CHK1 and CHK2, respectively [1, 2, 6–8]. In addition to these core pillars of the DDR, a third checkpoint effector pathway, mediated through TAO- and p38-dependent MK2 activity, was recently identified [9–12]. The p38/MK2 pathway is a global stress-kinase pathway that operates in parallel to CHK1. In response to DNA damage, this pathway is recruited as part of the ATM/ATR-dependent checkpoint network [11–14]. CHK1 and MK2 control checkpoint initiation and maintenance, respectively [15]. The activity of both kinases converges on mediating inhibitory phosphorylations on CDC25 family members [10, 16–18]. Specifically, CHK1- and MK2-dependent CDC25B phosphorylation on Ser-323 leads to the generation of a 14-3-3 binding epitope [10, 19, 20]. Upon 14-3-3 engagement, CDC25B is sequestered into the cytoplasm, away from its nuclear CDK substrates [21]. Thus, CHK1 and MK2 mediate inactivation of CDC25B and induce a cell cycle arrest by blocking CDC25B-dependent CDK activation [5].

Recently, the intimate connection between the DDR and post-transcriptional control of gene expression was highlighted through a proteomic analysis that identified proteins phosphorylated by the proximal DDR kinases ATM and ATR [22]. This screen revealed ~700 substrates, most of which belonging to pathways implicated in RNA metabolism [22]. Another proteomic analysis quantifying DNA damage-regulated alterations of the proteome, phosphoproteome and acetylome in response to etoposide resulted in a significant fraction of alterations involved in RNA metabolism [23].

Interestingly, a large number of RNA binding proteins (RBPs) have been shown to be directly or indirectly regulated by MK2 [5, 9–12, 24, 25]. Acting downstream of ATM and ATR, the p38/MK2 module is required to prevent G_1_/S, intra-S phase and G_2_/M transition after cisplatin and doxorubicin treatment in p53-deficient cells [11]. Following DNA damage, the p38/MK2 complex is exported to the cytoplasm, where it phosphorylates several RBPs, including PARN, TIAR and hnRNP A0 [15, 24, 26]. As a result of these phosphorylation events, the common mRNA client Gadd45a is stabilized leading to an increased expression of GADD45A protein levels, ultimately maintaining the p38/MK2 module in an active state through a positive feedback loop [15, 24, 26]. In addition to its role in maintaining p38/MK2 activity, GADD45A has also been shown to be a potent CDK inhibitor [27, 28].

Here, we show how label-free LC/MS/MS can be used for the profiling of functional RBP-mRNA interactome changes in response to genotoxic stress induced by etoposide. We further demonstrate how known RBP-mRNA interactions can be used together with transcriptomics to infer RBP activity. We show how changes in one regulator of RBPs, Mk2, can affect the overall spectrum of protein-RNA interactions in response to etoposide and how the RBP KHSRP contributes to DDR signaling through the regulation of *Cdkn1a* ^*P21*^.

## Materials and Methods

### Cell culture

Mouse embryonic fibroblasts (MEFs) were isolated as described previously [29], and cultured in high-glucose DMEM supplemented with 10% heat-inactivated FBS, 1% HEPES, 100 U/mL penicillin, and 100 μg/mL treptomycin (Gibco). Cells were treated with either etoposide (Sigma, E1383) or DMSO (vehicle / mock control; Carl Roth, A994.2). Mk2/3 knock out animals were previously published and were a kind gift from Matthias Gaestel at the Hannover Medical School [30]. *Khsrp* ^*-/-*^ MEFs were previously published and were a kind gift from Ching-Yi Chen at the University of Alabama at Birmingham [31]. Colony formation assays were performed as previously described [11]. Animal keeping was authorized by the “Landesamt für Natur, Umwelt und Verbraucherschutz Nordrhein-Westfalen” with the license 87–51.04.2010.A006.

### RNA isolation, sequencing, and qPCRs

RNA isolation was done using TRIzol reagent (Invitrogen, 15596–026) in agreement with manufacturer instructions. For RNA-sequencing RNA was further purified using a TruSeq Stranded Total RNA LT kit (with Ribo-Zero Human/Mouse/Rat). 1ug of total RNA was used as input material and hybridization-based negative enrichment of ribosomal sequences was performed. Afterwards, RNA fragmentation took place using divalent cations under elevated temperature. The RNA fragments underwent reverse transcription using random primers. This was followed by second strand cDNA synthesis with DNA Polymerase I and RNase H. To obtain strand specificity, a modified base is incorporated in this step. After end repair and A-tailing, indexing adapters were ligated, and the strand containing the modified base was digested. The products were then purified and amplified (15 PCR cycles) to create the final strand specific cDNA libraries. After validation (Agilent 2200 TapeStation) and quantification (Invitrogen Qubit System) we pooled 8 trancriptome libraries each. The pools were quantified by using the Peqlab KAPA Library Quantification Kit and the Applied Biosystems 7900HT Sequence Detection System. One pool was loaded on one lane of a Hiseq2000 sequencer and sequenced with a 2x100bp v3 protocol. We produced 4,1–6.5Gb/sample (41M-65M read paires). Basic read quality check was carried out using FastQC showing 86–90% of Q30 bases (PF) and a mean quality score between 34.1–35.2 (PF). Differential gene and transcript expression analysis was done using TopHat and Cufflinks [32]. Data can be accessed at the Gene Expression Omnibus under the access number GSE67266. Influence plots were done using the data contained on “The Atlas of UTR Regulatory Activity (AURA)”[33] and human and mouse homology tables from the mouse genome database [34]. Ontology analysis was done using DAVID [35, 36]. For real-time quantitative PCR (RT-qPCR), reverse transcription was performed using SuperScript VILO cDNA Synthesis Kit (Life Technologies, #11754050). RT-qPCRs were performed using Power SYBR Green (Life Technologies, #4367659) on an AB 7300 Real Time PCR System (Life Technologies). *Cdkn1a* forward primer: CTA TCA CTC CAA GCG CAG AT; *Cdkn1a* reverse primer: gca gcg tat ata cag gag acg; *Gapdh* forward primer: CCA ATG TGT CCG TCG TGG ATC T; *Gapdh* reverse primer: GTT GAA GTC GCA GGA GAC AAC C.

### Protein-RNA interactome assay

Protein-RNA crosslinking, and protein purification was performed as previously described [37]. For each replicate 4.0x10^8^ cells in 40 15cm dishes were UV irradiated at a wavelength of 254 nm with 200mJ/cm^2^ using a Bio-Link BLX 254 (peqLab). 250μg of protein were disgested in solution using trypsin. Afterwards, 1μg of peptide per sample was analyzed by nano LC/MS/MS with a Waters NanoAcquity HPLC system interfaced to a ThermoFisher Q Exactive. Peptides were loaded on a trapping column and eluted over a 75μm analytical column at 350nL/min; both columns were packed with Jupiter Proteo resin (Phenomenex). A 4h gradient was employed. The mass spectrometer was operated in data-dependent mode, with MS and MS/MS performed in the Orbitrap at 70,000 FWHM and 17,500 FWHM resolution, respectively. The fifteen most abundant ions were selected for MS/MS. Data were processed through MaxQuant software [38, 39]. Protein-protein based network expansion was performed using GeneMANIA [40].

### Cell cycle analysis, immunostainings

Cell cycle analysis was done using a Gallios flow cytometer (Beckman Coulter) and PI. After treatment, cells were washed twice in ice-cold PBS, trypsinized and fixed in 90% Methanol overnight at -20°C, permeabilized with PBS containing 0.25% Triton X-100 for 20 min at 4°C, blocked with 10% BSA in PBS and incubated with 1 μg of anti-phospho-histone H3 (Millipore, #16–218) or anti-gammaH2AX (Abcam, ab22551) per 10^6^ cells for 60 min on ice. Following washing, cells were incubated with FITC-conjugated secondary antibody (Millipore, #12–506) for 30 min on ice, washed, and resuspended in PBS containing RNase and 50 μg/ml PI prior to analysis.

### Immunoprecipitations and Western blotting

For RNA immunoprecipitations (RIP) protein-RNA crosslinking was performed as described above. Protein extraction was performed using cell lysis buffer in agreement with manufacturer instructions (Cell Signaling Technology, 9803) supplemented with RNase inhibitor (Thermo Scientific, #EO0381). Dynabeads Protein G Immunoprecipitation Kit was used in agreement with manufacturer instructions (Life Technologies, #10007D). 50 μl Dynabeads were incubated 4μg anti-Khsrp antibody (Biomol, #A302-021A) in a final volume of 200μl. After 30’ incubation at room temperature (RT) and rotating, supernatant (SN) was removed and 200μg protein added to a final volume of 900μl. Samples were incubated overnight, rotating, and washed with 1ml IP wash buffer (50mM HEPES-KOH, pH 7.5; 300mM KCl; 0.05% (v/v) NP40; 0.5mM DTT). Precipitates were then washed twice with high-salt wash buffer (50mM HEPES-KOH, pH 7.5; 500 mM KCl; 0.05% (v/v) NP40; 0.5mM DTT), resuspended in 1ml IP wash buffer and transferred to a clean reaction tube. After two PBS washes, RNA was eluted by incubation for 1h and 37°C with elution buffer (10mM Tris-HCl, pH 7.5; 1mM EDTA; 1% SDS; 4 mg/ml proteinase K). Afterwards, 400μl RNA phenol (Ambion, #9710) and 130μl of CHCl3 were added and standard phenol/chloroform RNA extraction performed. After collection of the aqueous phase 50μl of 3M NaOAc pH 5.2, 0.5μl of glycogen (Ambion, #9510) and 1ml of 1:1 EtOH:Isopropanol solution was added for overnight RNA precipitation and -20°C. After 10’ at 12000 G RNA pellets were washed twice with 500μl cold 75% EtOH, pellet dried and resuspended in 20μl H_2_O for 10’at 58μC. RT-qPCRs were performed as described above. Total amounts of *Cdkn1a* ^*P21*^ were normalized to whole Gapdh levels. Standard Western blotting was performed using the following antibodies: anti-P21, Santa Cruz Biotechnology, #sc-397; anti-Khsrp, Biomol, #A302-021A; anti-**β**-Actin, Sigma, #A5316.

## Results

### Etoposide-induced protein-RNA interactome changes

**Fig 1:**
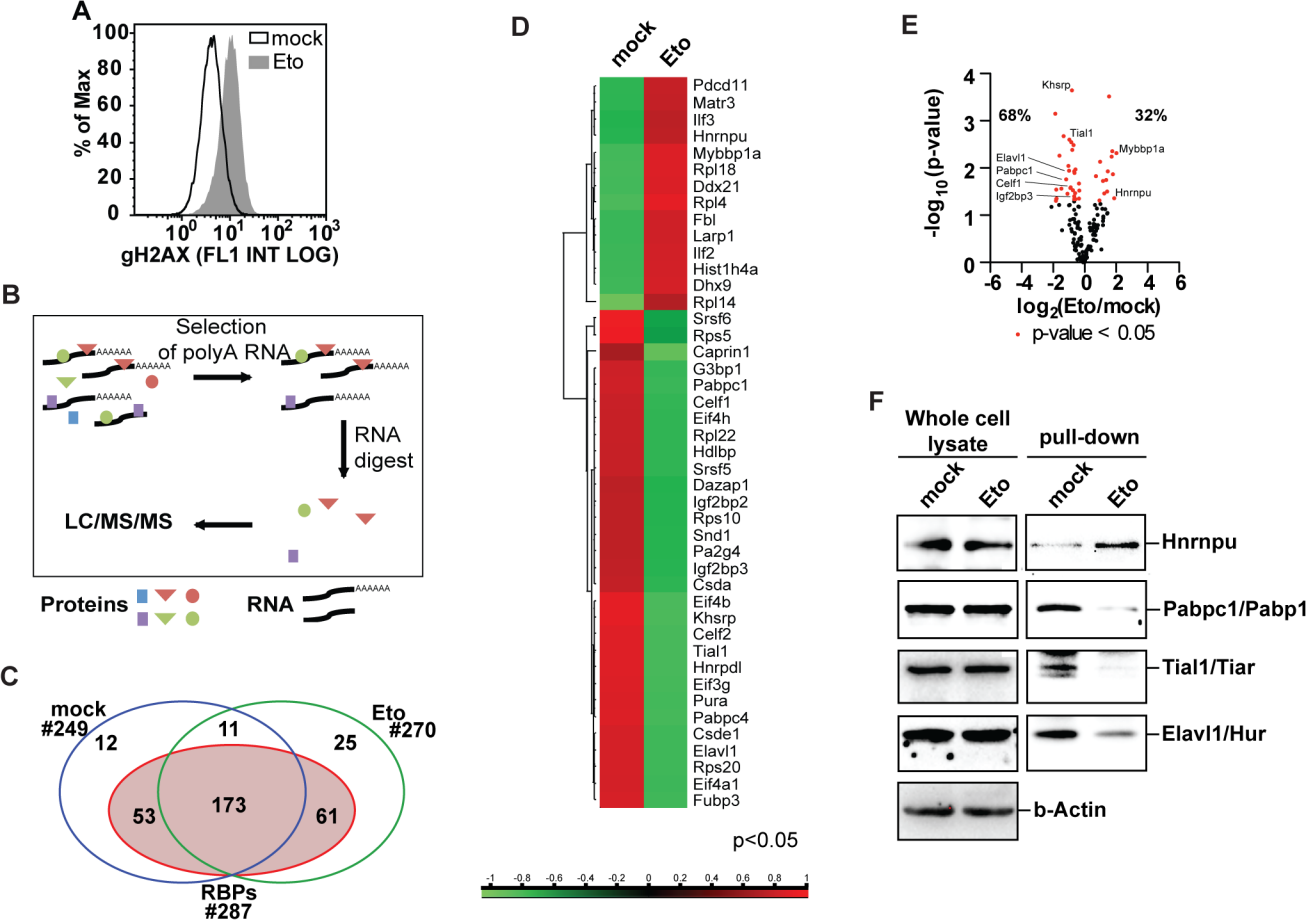
Changes in protein-RNA interactions in response to etoposide treatment. **(A)** Etoposide induces DNA double strand breaks after 6h of treatment as reported by the DSB marker **γ**-H2AX. **(B)** Schematics of the experimental procedure for the purification of RBPs show how UV-mediated crosslinking of RNA to interacting proteins was followed by poly-A selection to identify RBPs through LC/MS/MS. **(C)** Crosslinking followed by purification of mRNA-interacting proteins and nano LC/MS/MS identified 335 protein group hits of which 287 were known as RBPs. **(D)** Heat map of differentially abundant RBPs. **(E)** Vulcano plot representing changes in mRNA-protein interactions in response to etoposide treatment identifies Khsrp as the most significantly changed RBP in response to etoposide treatment. **(F)** Immunoblot analysis of proteins co-purified with poly-A-containing RNA validates protein-RNA interactome changes identified by label free LC/MS/MS (right panel). Whole cell lysates show no significant changes in protein levels of analyzed RBPs (left panel).

Mounting evidence for a role of RBPs in the DDR, as wells as the growing number of identified RBPs led us to the characterization of the changes in the protein-RNA interactome in response to DNA damage. For this purpose, primary MEFs from 13.5 days old embryos were treated with the DSB-inducing topoisomerase-II inhibitor etoposide (20μM). The occurrence of etoposide-induced genotoxic stress was validated using flow cytometry-based detection of histone H2AX phosphorylation at Ser-139. (**Fig 1A**). After 6h of treatment, RNA was covalently linked to its interacting proteins by UV-induced crosslinking. After lysis under denaturing conditions for removal of indirectly bound proteins, RBPs and poly-A-containing RNAs were co-purified using oligo-dT-coated magnetic beads (**Fig 1B**). Co-purified proteins were subsequently submitted to label-free quantification LC/MS/MS. Of the 335 obtained protein group hits, 287 contained proteins previously identified as RBPs (**Fig 1C**). Of the 184 RBPs present in both the untreated and etoposide-exposed samples, 44 RBPs were significantly altered in their abundance with RNA following etoposide (**Fig 1C–1E**). We next validated these mass spectrometry-derived differences using immunoblotting. As shown in **Fig 1F**, protein levels in whole cell lysates showed no change in response to etoposide treatment, while the etoposide-induced changes that were observed in the RNA-bound fractions could be reproduced by immunoblotting. These data suggest that the interaction between the RBPs under investigation and their client mRNAs is indeed altered after genotoxic stress (**Fig 1F**, left panel). Further corroborating this hypothesis, transcript levels of RBPs showing differential protein-RNA interaction, were also not changed (**S3 Table**). Thus, etoposide treatment induces protein-RNA interactome changes that can be identified by label-free LC/MS/MS and validated by immunoblotting. Interestingly, changes in protein-RNA interactions do not correlate with changes in overall protein levels.

### Transcriptomics-based identification of differential RBP-client interactions

**Fig 2:**
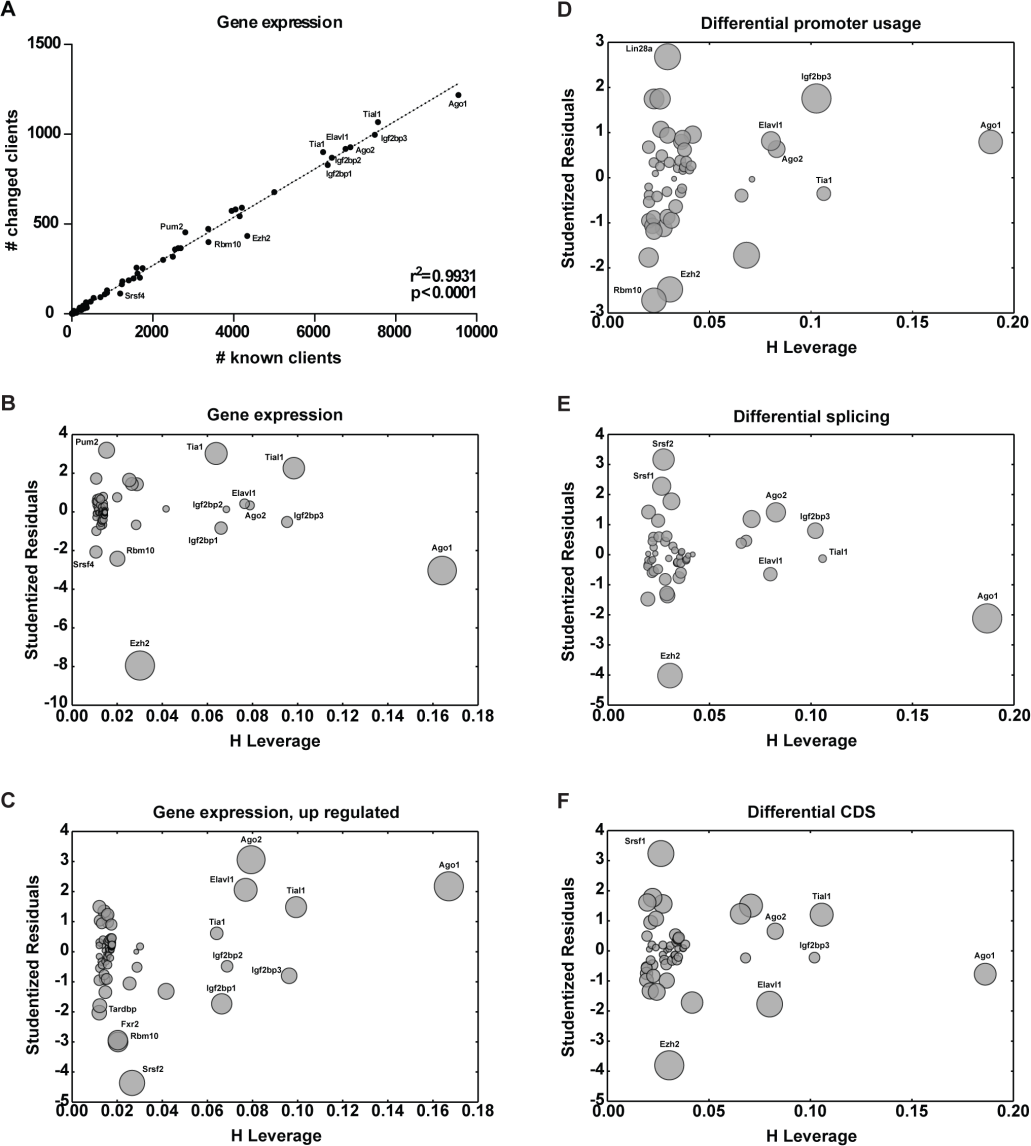
Inference of outlying RBP-client interactions from changes in client transcripts. **(A)** Using gene expression levels, numbers of altered client mRNAs were plotted against number of known client mRNAs for each respective RBP. A linear correlation could be identified between the number of changed client mRNAs and known client mRNAs. **(B)** Studentized residuals (outlyingness), leverage (potential to influence the linear model) and influence analysis (represented by the size to point) are represented through influence plots. Data points perturbing the model were identified by high leverage and studentized residuals. Outliers representing RBPs with higher number of changed client mRNAs were identified through high absolute values of standardized residuals. The same was done by **(C)** plotting number of upregulated clients against number of changed clients, as well as using vector information on **(D)** differential promoter usage, **(E)** differential splicing, and **(F)** differential CDS. DNA damage-related RBPs—Elavl1, Tia1, Tial1, Srsf1, Srsf2 could be identified through RBP-client analysis.

We next hypothesized that changes in the transcript levels of client mRNAs are a result of differential RBP activity. For this purpose, cells were either treated with etoposide (20μM, 6hr) or exposed to vehicle control. Upon completion of drug exposure, cells were harvested, RNA was isolated and transcriptome analysis was performed using RNA-seq. We then asked how the number of altered mRNA clients for each RBP is correlated to the number of respective known mRNA clients (**Fig 2A**). Regression analysis identified a linear correlation between the number of known client RNAs and the number of client mRNAs with altered overall expression (known vs. changed; R^2^ = 0.9931, p<0.0001, **Fig 2A**). Outlying points identified RBPs associated with stronger changes in client mRNA expression (**Fig 2A**). Intriguingly, Ago1 emerged as an outlier and as the RBP with the largest number of known targets (**Fig 2A**). Thus, the large number of known Ago1 clients isolates this point from the remaining population making it difficult to assess its relation to the regression. Thus, isolation is better characterized by leverages—the potential for an individual data point to influence the entire model. As with Ago1, the more isolated a data point is, the stronger its potential to influence the model becomes. Cases like Ago1, outliers with a high leverage, have the highest influence on the model, perturb the model, and should be either excluded or taken into account with care. We therefore plotted studentized residuals (a measure of outlyingness [41]) in function of the leverage—influence plots (**Fig 2B**) [41]. Interestingly, Tial1, one of the RBPs identified in our interactome analysis emerged as an outlier (**Fig 2B**). Furthermore, correlation analysis between number of upregulated clients and number of clients with altered expression showed that most Tial1 targets were upregulated (**Fig 2C**). This is consistent with our interactome analysis showing Tial1 dissociation from RNA and the fact that Tial1 is a component of SGs where RNAs are sequestered until degraded or released once a stress response is terminated [42].

Transcripts produced by RNA polymerase II (PolII) are subject to multiple processing steps, including maturation of 5’ and 3’ ends and splicing, followed by transport to the cytoplasm [43–48]. Correlation analysis involving the different steps of RNA maturation was revealing of the above-introduced roles of Srsf1 and Srsf2 in the regulation of differential splicing in response to etoposide (**Fig 2E and 2F**) [43–48]. To further understand the orchestration of post-transcriptional events involved in the DDR, we investigated the possibility of different transcripts being targeted to RBPs by transcriptional activation dependent on differential promoter usage (**Fig 2D**). Interestingly, Lin28a, Ezh2, and Rbm10 emerged as outliers suggesting a role for differential promoter usage of client transcripts in the function of these RBPs. Together with transcriptome analysis of RBPs and protein-RNA interactome analysis, our transcriptomic based analysis of RBP activity contributes to characterization of the RBP-mediated response to etoposide treatment.

### Transcriptome and interactome changes in etoposide-driven G_2_/M arrest

**Fig 3:**
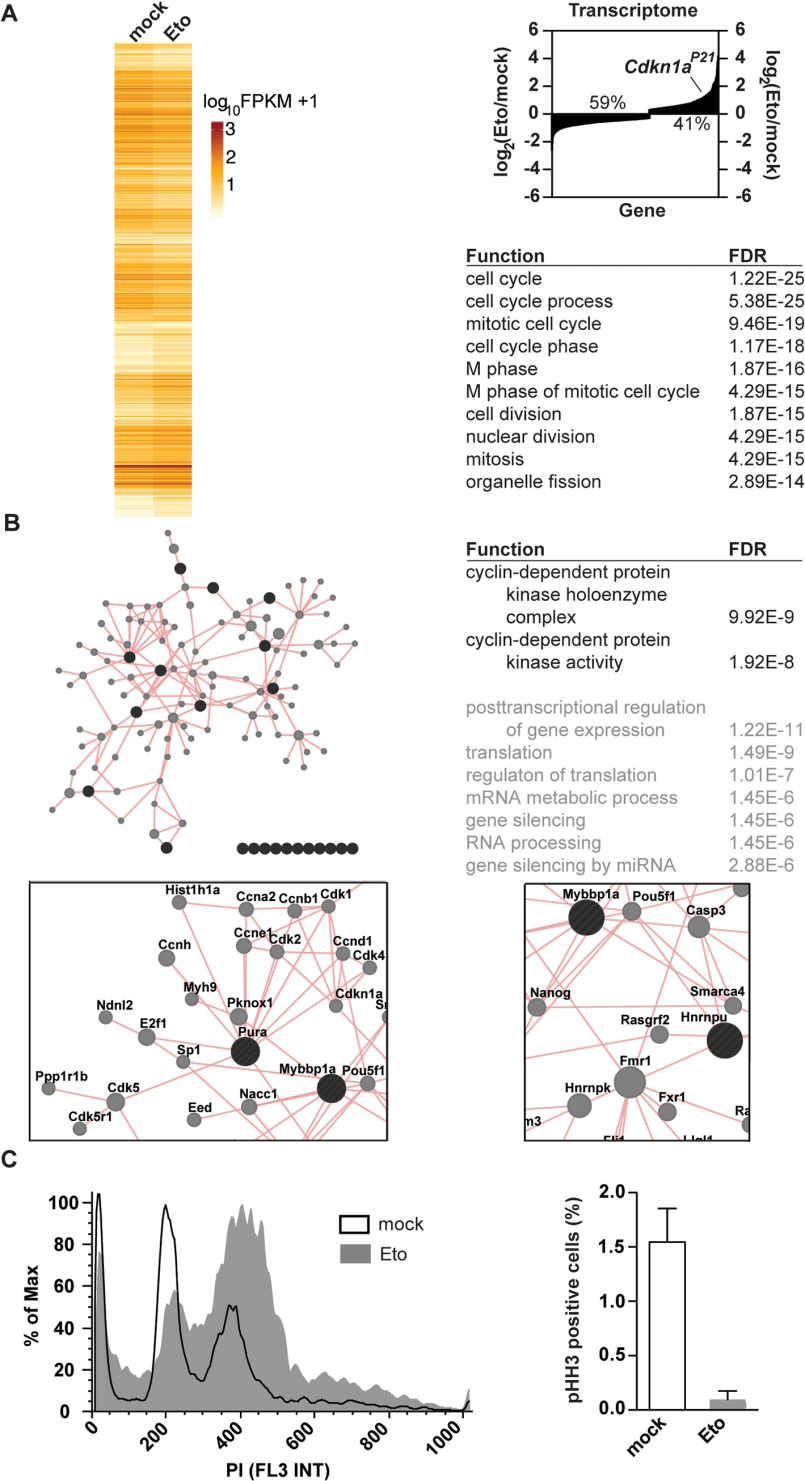
Murine embryonic fibroblasts arrest in G_2_ in response to etoposide treatment. **(A)** Gene expression changes identified by RNA-Seq following 6h of treatment with 20μM etoposide were analyzed for enrichments in GO terms. Cell cycle, and specifically mitotic processes, emerged in the top 10 most significant GO terms. **(B)** Protein-protein interactions-based network expansion of RBPs showing differential protein-RNA interactions upon etoposide treatment identifies enrichments for cyclin-dependent processes. **(C)** Cell cycle analysis of untreated (black line) and etoposide-treated (gray) cells reveals an accumulation of cells with 4N DNA content and decreased staining of the mitotic marker pHH3.

To understand how protein-RNA interactome changes, as well as transcriptome alterations correlate to global changes in cellular processes, we performed gene ontology (GO) enrichment analysis. GO annotations offer the largest collection of process-associated terms for any related gene [49]. To identify the processes being changed in response to etoposide treatment, we performed GO analysis on the list of genes showing significant gene expression changes in response to etoposide treatment. With 37,981 genes being read, 13,508 could be tested for differential expression. While only a limited number of alterations in gene expression could be observed after 1h of treatment (104 genes with p<0.05 and 59 genes with q<0.05), the expression of 1,808 genes was significantly changed after 6h of etoposide treatment (p<0.05, **Fig 3A**, 771 genes with q<0.05). In agreement with the known role and upregulation of *Cdkn1a* ^*P21*^ in the DDR, *Cdkn1a* ^*P21*^ was transcriptionally upregulated following etoposide treatment (2.2 fold, p = 5.0x10^-05^). Probably not surprising, GO term analysis revealed a significant enrichment for transcripts involved in cell cycle regulation among the genes with altered expression after etoposide exposure (**Fig 3A**). We then investigated the possibility of protein-protein interactions being the basis of the observed interactome changes. To directly address this, we used GeneMANIA [40] to perform protein-protein based network expansion of the 44 differentially abundant RBPs using linear regression to automatically choose genes that promote the highest number of interactions. GO analysis of the expanded network identified cyclin-dependent protein processes amongst the most significantly enriched processes (**Fig 3B**). In agreement with this Cdkn1a^P21^, as well as Cdk1, Cdk2 and Cdk4 emerged as part of the generated network. (**Fig 3B**). These data connect RBPs to cell cycle regulation during the response to etoposide treatment.

In agreement with the enrichment of GO terms emerging from transcriptome and interactome analysis, etoposide treatment of MEFs (20μM, 24hr) resulted in an accumulation of phospho-histone H3-negative cells with 4N DNA content, indicating a G_2_/M arrest (**Fig 3C**). Furthermore, primary MEFs treated with etoposide showed a significant drop of the mitotic index (**Fig 3C)**. Transcriptome and interactome profiling emerged therefore as two useful tools for the characterization of changes involved in the etoposide-driven G_2_/M arrest.

### Mk2/3-mediated regulation of RBPs

**Fig 4:**
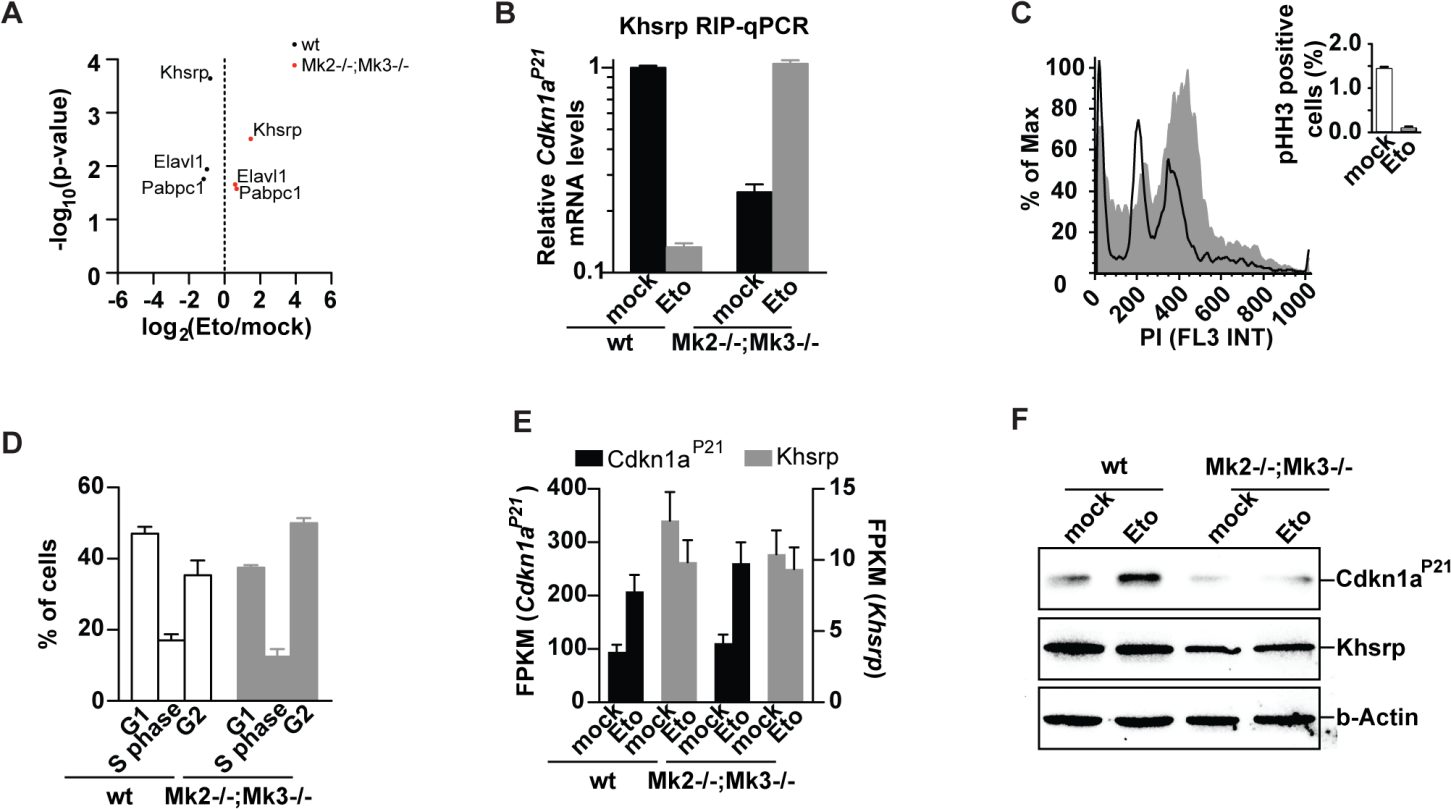
Mk2/3-dependent regulation of Khsrp and *Cdkn1a* ^*p21*^. **(A)** *Mk2* ^***-/-***^ *;Mk3* ^***-/-***^ cells show a differential protein-RNA interactome in response to etoposide exposure when compared to wildtype cells (most-significant changes are highlighted). **(B)** *Khsrp* RNA immunoprecipitations (RIP) followed by *Cdkn1a* ^***P21***^ qPCR validates interactome changes seen in wildtype and *Mk2* ^***-/-***^ *;Mk3* ^***-/-***^ cells upon etoposide treatment. Upon etoposide exposure Khsrp is released from *Cdkn1a* ^***P21***^ transcripts. In contrast, in *Mk2* ^***-/-***^ *;Mk3* ^***-/-***^ MEFs, Khsrp-bound *Cdkn1a* ^***P21***^ transcripts increase upon etoposide exposure. **(C)** Despite a typical arrest in G_**2**_ upon etoposide treatment, **(D)** *Mk2* ^***-/-***^ *;Mk3* ^***-/-***^ MEFs show a decreased G_**1**_ population in comparison to wt cells. **(E)** Increased levels of the *Cdkn1a* ^***P21***^ transcript in *Mk2* ^***-/-***^ *;Mk3* ^***-/-***^ cells upon etoposide treatment **(F)** fail to promote the upregulation of Cdkn1a^***P21***^ protein levels seen in wildtype cells.

We and others have shown that the checkpoint effector kinase pathway that is governed by p38**α**/**β**-dependent activation of MK2 is activated in response to DNA-damaging agents, such as UV and different chemotherapeutic drugs [5, 9–12, 24, 25]. Interestingly, our protein-RNA interactome analysis revealed that the known p38 targets, Elavl1 [50], Khsrp [51], Tial1 [25], as well as the MK2 target Pabpc1 [52], are differentially bound to RNA in response to etoposide. To directly investigate the impact of the p38/Mk2 kinase complex on the RBP-RNA interactome, we isolated MEFs from *Mk2/3* knock-out (KO) mice and investigated the interactome changes in response to etoposide treatment (**Fig 4A**). In contrast to wildtype cells, the p38- and Mk2 targets, Elavl1, Pabpc1 and Khsrp could be co-purified in higher amounts with poly-A-containing RNA once *Mk2/3* KO cells were treated with etoposide (**Fig 4A**, p<0.05). Thus, Mk2/3 depletion inverted the protein-RNA interactions seen for p38/Mk2 targets in wildtype cells upon exposure to etoposide (**Fig 4A**). Furthermore, in the absence of Mk2/3, the p38 target Tial1 did not change its interaction with RNA, as previously observed in wildtype cells (**S2 Table**). Transcriptomics based inference of RNA-protein interactions suggested a role for the Mk2-target Elavl1 in the DDR (**Fig 2**, **S1 and S2 Figs**). The RBP Khsrp has been previously shown to post-transcriptionally regulate a variety of AU-rich elements (AREs) [51, 53]. With four contiguous K homology (KH) motifs driving the recognition of the AREs, KHSRP further interacts with the exosome and PARN [53]. Intriguingly, binding of KHSRP to its client mRNAs has been shown to be negatively regulated by p38 [51]. Of note, the transcript encoding the CDK4 and -6 inhibitor Cdkn1a^P21^ recently emerged as a Khsrp target [51]. In agreement with this, we found that Khsrp co-precipitates with *Cdkn1a* ^*P21*^ mRNA in untreated wildtype cells, while this interaction appears to be disrupted upon etoposide treatment (**Fig 4B**). In marked contrast, etoposide exposure did not lead to a dissociation of the Khsrp-*Cdkn1a* ^*P21*^ mRNA complex in *Mk2/3* KO cells (**Fig 4B**). Furthermore, lower levels of *Ckdn1a* ^*P21*^ mRNA bound to Khsrp were observed in non-treated *Mk2/3* KO cells, when compared to untreated wildtype cells. Cell cycle analysis revealed that despite a functional G_2_/M arrest in response to etoposide treatment (indicated by a stably repressed mitotic index), *Mk2/3* KO cells display a decreased G_1_ and increased G_2_ population, when left untreated (**Fig 4C and 4D**). Thus, *Mk2/3* KO MEFs display a defect in cell cycle regulation that either promotes a faster progression through G_1_ or a slower progression through G_2_. In agreement with the latter, hematopoietic stem cells derived from *Mk2* KO mice have been previously shown to display increased proliferation rates and a diminished G_1_ population [54]. As a faster progression through G_1_ can be the result of lower Cdkn1a^P21^ levels, we investigated the changes in mRNA and protein levels of *Cdkn1a* in wildtype and *Mk2/3* KO cells (**Fig 4E and 4F**). Interestingly, while mRNA levels in *Mk2/3* KO cells fully copied the values observed in wildtype cells, *Mk2/3* KO cells showed decreased Cdkn1a^P21^ protein levels and a complete lack of Cdkn1a^P21^ upregulation in response to genotoxic stress (**Fig 4E and 4F**). Thus, in agreement with the changes in protein-RNA interactions seen upon *Mk2/3* deletion, transcript and protein levels of Cdkn1a^P21^ appear to be uncoupled in *Mk2/3* KO cells further suggesting a role for RBPs in the translational control of Cdkn1a^P21^. These results add another piece of evidence to the notion that *Cdkn1a* ^*P21*^ is post-transcriptionally regulated in response to DNA damage and particularly etoposide [24, 55].

### KHSRP-mediated control of cell cycle

**Fig 5:**
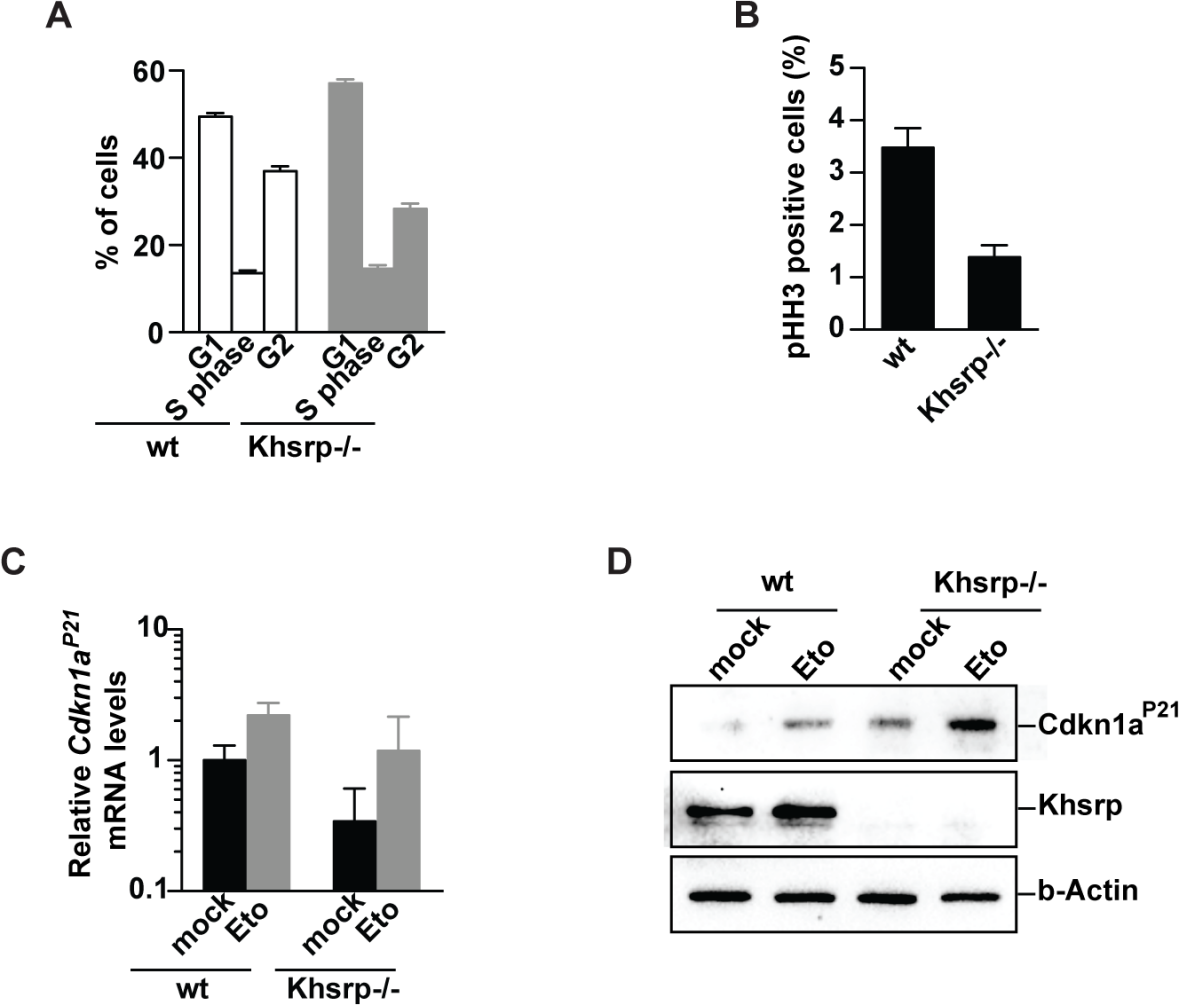
Khsrp-dependent regulation of *Cdkn1a* ^*P21*^. **(A)** Cell cycle analysis and **(B)** mitotic index of wildtype and *Khsrp* ^***-/-***^ MEFs expose a *Khsrp*-dependent **(A)** accumulation of cells in G_**1**_ and **(B)** decreased cycling rates. **(C)** Transcript and **(D)** protein levels of *Cdkn1a* ^***P21***^ in *Khsrp* ^***-/-***^ cells measured by qPCR and immunoblotting reveals an increase of Cdkn1a^***P21***^ protein levels unrelated to *Cdkn1a* ^***P21***^ mRNA levels.

Cells with a deficient p38/Mk2 module show an overall altered interaction between Khsrp and poly-A-containing RNAs (**Fig 4A**). Interestingly, such *Mk2/3* KO cells have decreased G_1_ populations and lower protein levels of the G_1_ regulator and Khsrp client Cdkn1a^P21^ (**Fig 4F**). To further investigate the role of Khsrp in cell cycle regulation, we performed cell cycle analysis of *Khsrp*-deficient MEFs (**Fig 5A**). *Khsrp* KO MEFs showed an increase in G_1_ population and lower mitotic index, indicative of a reduced proliferation rate due to a delayed G_1_/S transition (**Fig 5A and 5B**). In agreement with this, Cdkn1a^P21^ protein levels were higher in *Khsrp* KO than in wildtype cells. Interestingly, transcript levels of *Cdkn1a* ^*P21*^ remained equal between *Khsrp* KO and wt cells (**Fig 5C and 5D**). Despite a delayed G_1_/S transition *Khsrp* KO cells do arrest in G_2_ upon exposure to etoposide (**S3 Fig**). Nonetheless, a significant etoposide resistance can be seen in *Khsrp* ^*-/-*^ cells (**S4 Fig**). These results outline the relevance of Khsrp in the post-transcriptional regulation of Cdkn1a^P21^ and control of the cell cycle.

### KHSRP as and indicator of overall survival and disease-free survival

**Fig 6:**
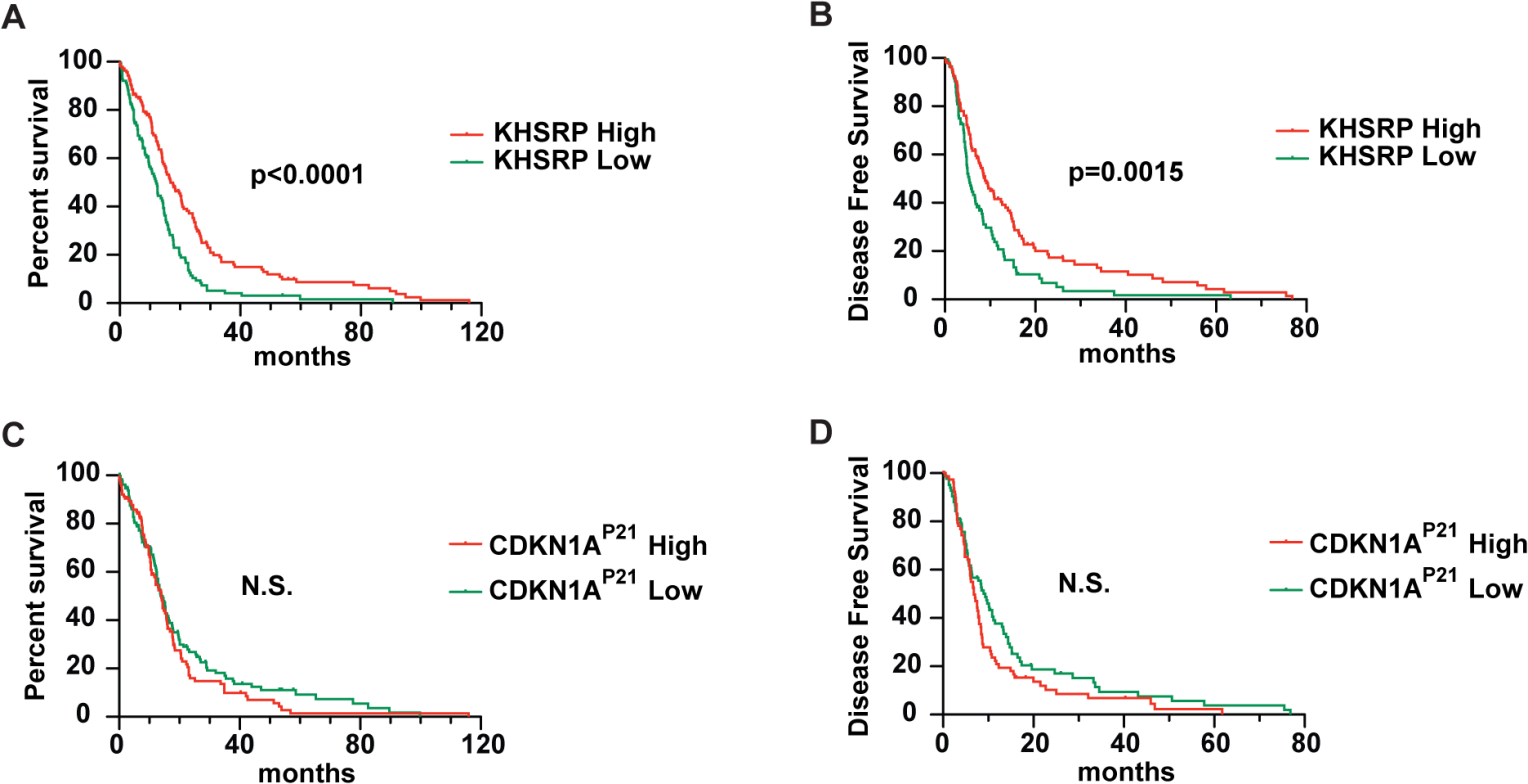
*KHSRP* transcript levels predict survival of human glioblastoma patients. **(A)** Overall survival (OS) curves and **(B)** disease free survival (DFS) curves show an increased OS and DFS of patients bearing tumors with higher *KHSRP* transcript levels. **(C,D)** Although not significant, the inverted tendency can be seen when segregating patients in agreement with their tumor *CDKN1A* ^***P21***^ transcript levels. Upper and lower quartiles are shown.

The transformation of a normal cell into a cancerous cell is often the result of altered function of proteins involved in cell division [56]. A crucial example is the inactivation of *TP53*, a transcriptional activator of the cell cycle regulator *CDKN1A* ^*P2*1^ [57]. It is than plausible to hypothesize that other *CDKN1A* ^*P21*^ regulators might also have a role in malignancy. Thus, we pursued the possibility of an association between the *CDKN1A* ^*P21*^ post-transcriptional regulator KHSRP and known malignancies. To this end, we recurred to the “cBioPortal for Cancer Genomics” to extract data generated by “The Cancer Genome Atlas Research Network” (TCGA) [58, 59]. Gene expression data for cases with associated clinical data revealed that *KHSRP* expression can be used as an indicator of overall survival (OS) and disease free survival (DFS) for glioblastoma multiforme patients (**Fig 6A and 6B**). Patients bearing tumors with lower levels of *KHSRP* transcripts showed a decreased OS consistent with a decreased DFS (p<0.0001 and p = 0.0015). In agreement with the increased G_1_ population and reduced mitotic index in *Khsrp* KO cells (**Fig 5B**), tumors with reduced *KHSRP* expression might display higher levels of the CDKN1A^P21^ protein and delayed G_1_ progression. However, when using *CDKN1A* ^*P21*^ gene expression as a stratifier, OS and DFS were not significantly different between those patients that expressed high levels of *CDKN1A* ^*P21*^ and this with reduced expression (**Fig 6C and 6D**). We note that KHSRP is regulating not only *CDKN1A* ^*P21*^ mRNA levels, but controls a plethora of different target genes [60]. We thus speculate that the net effect of KHSRP loss might not phenocopy the loss of *CDKN1A* ^*P21*^. Although we could not observe the same significance levels in the current available data for other tumor entities (**S7 Table**), the indicative power of *KHSRP* in DFS and OS in glioblastoma multiforme further suggests a strong role for this gene in the maintenance of genome stability.

## Discussion

Mounting evidence collected over the last few years supports the idea that RBPs involved in different steps of mRNA biogenesis, translation and degradation can affect genome stability programs [22, 43, 61, 62]. Here, we employed oligo(dT) pulldowns to profile the changes in the protein-RNA interactome in primary MEFs upon exposure to etoposide (**Fig 1**). Despite the large number of protein hits identified by others in human cells lines (approx. 1,000 per study) [63–65], we were only able to identify 184 recurrent protein-groups in our interactome study (**Fig 1C**). While previous studies relied on immortalized human cell lines, we used freshly isolated primary MEFs. Another possible reason for the observed differences might be the presence of RNAse in our peptide mixture. While others removed RNAse from the protein mixtures by gel excision and selection of the fragments of interest, we have purposely omitted this step in favor of a higher accuracy of label-free quantification. Of the 335 identified hits, 47 were unique to our study (**Fig 1C, S1 Table**). Although further work is required to validate these proteins as RBPs our observation points towards a further expansion of the number of known RBPs to 1,549. While immunoblot validation fully supported our LC/MS/MS results, analysis of whole cell lysates revealed that for the studied proteins, etoposide-induced alterations in client mRNA binding do not result from changes in overall RBP expression levels (**Fig 1F**). Thus, the changes in interactome can be the result of changes in the expression patterns of the clients (target RNAs), or of signaling events promoting enhanced binding or dissociation from the client mRNAs. In agreement with the first, differential promoter usage revealed that the clients of Lin28a, Rbm10, and Ezh2 are expressed through different promoter sequences upon exposure to etoposide (**Fig 2D**). In support of the latter, protein-protein based network expansion of the significantly changed RBPs revealed that the expanded network is highly enriched for processes involving Cdk activity (**Fig 3B**).

Using RBP-client interactions catalogued within the AURA database [33], we identify RBPs with etoposide-induced outlying client interactions based on transcript alterations of the respective clients. As the number of known clients greatly differs between each RBP, we linearized the population by plotting the number of changed clients against the number of known RBPs (changed vs. known clients, **Fig 2A**). This *in silico* analysis allowed us to properly identify RBPs with outlying changes in client mRNA expression, while taking into account the amount of available information for each RBP and its influence on the regression analysis. Interestingly, this approach **(Fig 2**) identified several RBPs which also showed differential protein-RNA interactions on our label-free LC/MS/MS assay (**Fig 1D**): Tia1, Tial1, and Elavl1. The cross-occurrence of these hits over our interactome and transcriptomics-based analyses strongly enforces a role for these RBPs in the DDR. Intriguingly, Tial1 is known to bind to *Gadd45a* mRNA preventing its association with translating polyribosomes in steady state scenarios [66]. In response to genotoxic stress, translation of GADD45A is then enhanced by dissociation of TIAL1 from *GADD45a* mRNA [66]. Further, ELAVL1 is known to act as a post-transcriptional regulator in response to genotoxic stress and previous work has shown that UVC exposure results in *CDKN1A* ^*P21*^ mRNA stabilization through ELAVL1 [67].

MK2 phosphorylation by p38 in response to genotoxic stress drives the export of these two molecules from the nucleus, through the exposure of a nuclear export signal on Mk2 [25, 68]. While p38 phosphorylates ELAVL1 [50], KHSRP [51], and TIAL1 [25], MK2 phosphorylates PABPC1 [52], PARN [25], TTP [69], BRF1 [70], and hnRNP A0 [71]. Here we show that disruption of the p38/Mk2 complex through *Mk2* deletion results in strong protein-RNA interactome changes in response to genotoxic stress induced by etoposide (**Fig 4A and 4B**). In *Mk2* null cells, the p38 targets Khsrp and Elavl1, as well as the Mk2 target Pabpc11 become more abundant with poly-A-containing RNAs, compared to wildtype cells (**Fig 4A and 4B**). In agreement with this altered binding pattern, transcriptomics-based identification of outlying RBP-client interactions shows a decreased outlyingness of Elavl1 in *Mk2* depleted cells (**Fig 2, S1 and S2 Figs**). Upon phosphorylation by p38, KHSRP shows a decreased binding to the *Cdkn1a* ^*P21*^ transcript and other ARE-containing transcripts and fails to promote their rapid decay, while retaining its ability to interact with the mRNA degradation machinery [51]. In agreement with this, Khsrp becomes less abundant with poly-A-containing RNA (**Fig 1D**) and in particular with *Cdkn1a* ^*P21*^ mRNA, upon etoposide treatment (**Fig 4B**). Disruption of the p38/Mk2 module and continuous Khsrp binding to *Cdkn1a* ^*P21*^ mRNA was associated with low *Cdkn1a* ^*P21*^ protein levels uncoupled from the increase in the *Cdkn1a* ^*P21*^ transcript upon exposure to etoposide (**Figs 1D and 4B**).

CDKN1A^P21^ is a potent CDK inhibitor that binds to and inhibits the activity of CDK4 and 6, and thus functions as a regulator of cell cycle progression at G_1_ [72]. We here show that, *Mk2/3* KO MEFs have lower CDKN1A^P21^ protein levels and a decreased G_1_ population (**Fig 4F and 4D**). We further show that *Mk2* deletion affects the binding pattern of the post-transcriptional *Cdkn1a* ^*P21*^ regulator Khsrp (**Fig 4A and 4B**). This is most probably the result of nuclear retention of p38 in the absence of the nuclear export signal of Mk2. The need for p38 phosphorylation for Khsrp release of its client RNAs renders the p38/Mk2 module a negative regulator of Khsrp. In agreement with the lower G_1_ population in *Mk2*-deficient cells, *Khsrp* KO cells have an increased G_1_ population, increased CDKN1A^P21^ protein levels, and a decreased mitotic index (**Fig 5**). These results underscore the role of the p38/Mk2/Khsrp pathway in cell cycle regulation.

Maintaining genome stability is crucial for cell growth and cell survival. Different genetic disorders, including most human cancers, are associated with different forms of genome instability [57]. *Cdkn1a* ^*P21*^ regulation by the tumor suppressor p53 and the increasing evidence for a role of RBPs in genome stability [22, 43, 61, 62] suggested a role for the post-transcriptional regulator of *CDKN1A* ^*P21*^, KHSRP, in tumorigenesis. In contrast to p53, KHSRP is as negative regulator of *CDNK1A* ^*P21*^. Indeed, analysis of the data curated by “The Cancer Genome Atlas Research Network” (TCGA) [58, 59] revealed that high *KHSRP* transcript levels are associated with increased overall survival in glioblastoma multiforme (**Fig 6A**). This might be the result of a better response to therapy as high *KHSRP* levels were also associated with increased disease free survival (**Fig 6B**). The lack of power for *KHSRP* to predict OS and DFS in other entities might be the reflection of a different relevance of *KHSRP* in different tissues and entities, as wells as the result of lower data availability. Thus, tumor cells with high levels of KHSRP, might have a reduced capacity to survive genotoxic therapies due to defective G_1_ checkpoints.

## Conclusions

We have shown that RBPs have distinct binding patterns in response to genotoxic stress induced by etoposide. We show how differential RBP profiles can be identified using protein-RNA interactome approaches, as well as transcriptomics. In addition to validating the role of many known RBPs in the etoposide-induced DDR (e.g. Srsf1, Srsf2, Elavl1), we add a new collection of RBPs to the etoposide-induced DDR. We demonstrate how changes in one key RBP regulating signaling module, p38/Mk2, can affect the entire spectrum of protein-RNA interactions. We further validate Khsrp as a cell cycle regulator through the regulation of *Cdkn1a* ^*P21*^. Finally we identify KHSRP as a predictor of overall survival, as well as disease free survival in glioblastoma multiforme.

## Supporting Information

S1 Fig Inference of RBP activity from target levels on *Mk2* ^*-/-*^;Mk3^*-/-*^ cells. **(A)** Using gene expression levels, number of changed client mRNAs were plotted against number of known client mRNAs for each respective RBP. A linear correlation could be identified between the number of changed client mRNAs and known client mRNAs. **(B)** Studentized residuals (outlyingness), leverage (potential to influence the linear model) and influence analysis (represented by the size to point) are represented through influence plots. Data points perturbing the model were identified by high leverage and studentized residuals. Outliers representing RBPs with higher number of changed client mRNAs were identified through high absolute values of standardized residuals. The same was done by **(C)** plotting number of upregulated clients against number of changed clients, as well as using vector information on **(D)** differential promoter usage, **(E)** differential splicing, and **(F)** differential CDS.
(TIF)

S2 Fig Difference in outlyingness of RBPs between wildtype and *Mk2* ^*-/-*^;Mk3^*-/-*^ cells. Shown are the RBPs with the highest changes in RNA-protein interactome changes and for which enough information on client mRNAs changes was available.
(TIF)

S3 Fig Khsrp^-/-^ cells arrest in G_2_ in response to etoposide. Upon etoposide treatment, Khsrp^-/-^ MEFs arrest in G_2_ as seen by the increase in cells with a 4N DNA content and decrease in pHH3 positive cells.
(TIF)

S4 Fig *Khsrp* ^*-/-*^ cells display increased resistance against etoposide. Colony formation assay of etoposide-treated wt and *Khsrp* ^*-/-*^ cells reveals a significant resistance of *Khsrp* ^*-/-*^ cells to etoposide treatment. **(A)** Colony formation assays following a 12 hr exposure to 20μM etoposide are shown for wt and *Khsrp* ^*-/-*^ cells. **(B)** Quantification of the data shown in **(A)**. At least 6 high power fields were evaluated for this analysis. **(C)** Representative microscopic view of the cells shown in **(A, B)**. HPF, high power field; Eto, etoposide.
(TIF)

S1 Table Label-free quantification of protein-RNA interactome changes in wt MEFs upon 6h exposure to 20 μM etoposide. (XLSX)

S2 Table Differential gene expression analysis of wt and *Mk2/3* KO MEFs upon 1h and 6h exposure to 20 μM etoposide. (XLSX)

S3 Table Differential promoter usage in wt and *Mk2/3* KO MEFs upon 1h and 6h exposure to 20 μM etoposide. (GZ)

S4 Table Differential splicing in wt and *Mk2/3* KO MEFs upon 1h and 6h exposure to 20 μM etoposide. (GZ)

S5 Table Differential CDS in wt and *Mk2/3* KO MEFs upon 1h and 6h exposure to 20 μM etoposide. (GZ)

S6 Table Log-rank test results of overall survival Kaplan-Meier plots for clinical studies not showing significant differences between patients with high Khsrp and low Khrsp. (GZ)

S7 Table Log-rank test results of overall survival Kaplan-Meier plots for clinical studies not showing significant differences between patients with high Khsrp and low Khrsp. (CSV)
