# Supplementary figures and images for: Label-Free Protein-RNA Interactome Analysis Identifies Khsrp Signaling Downstream of the p38/Mk2 Kinase Complex as a Critical Modulator of Cell Cycle Progression
Source: PLoS One. 2015 May 20;10(5):e0125745. doi: 10.1371/journal.pone.0125745 (PMC4439058; doi:10.1371/journal.pone.0125745)

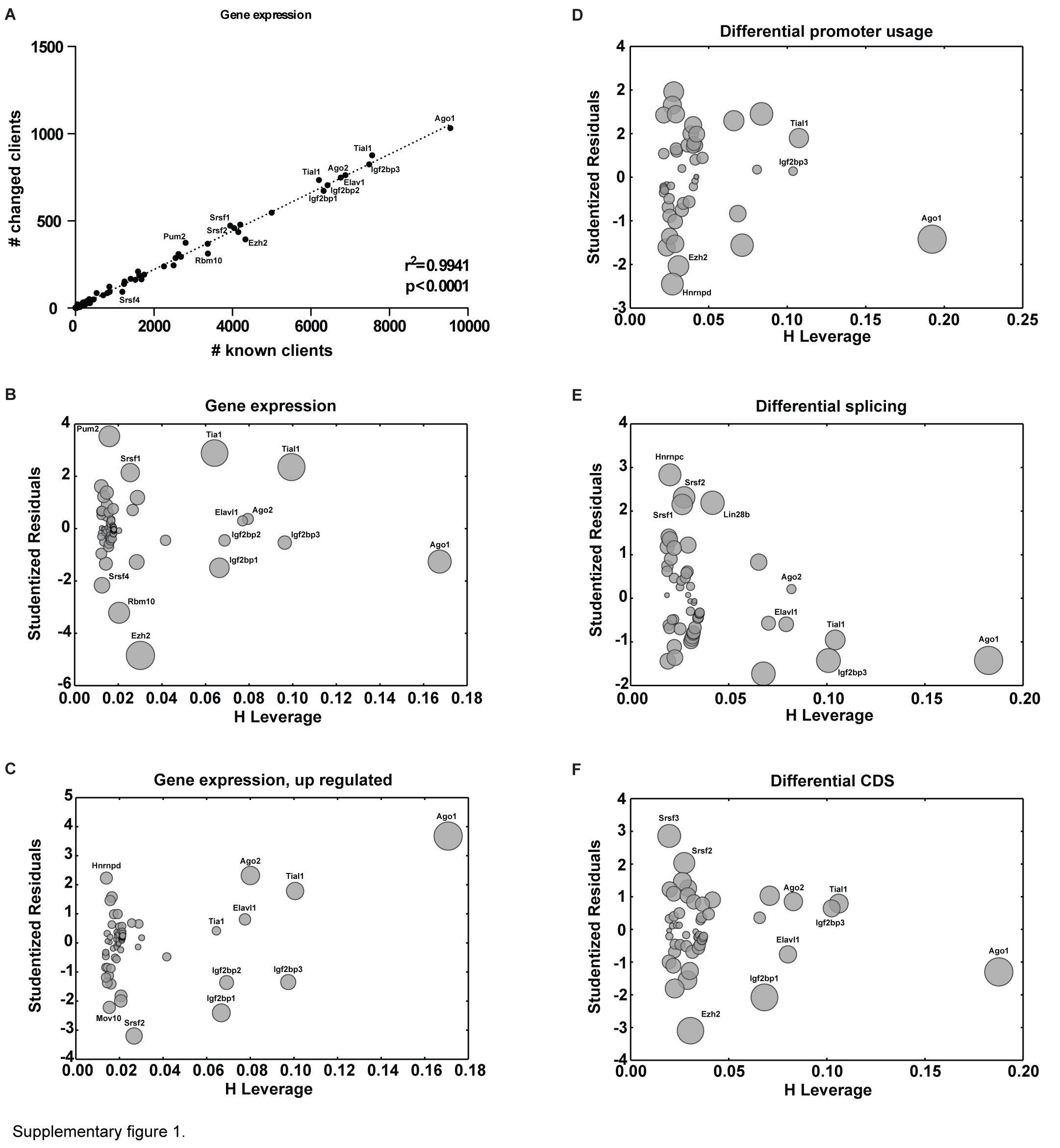

Supplement: S1 Fig — (A) Using gene expression levels, number of changed client mRNAs were plotted against number of known client mRNAs for each respective RBP. A linear correlation could be identified between the number of changed client mRNAs and known client mRNAs. (B) Studentized residuals (outlyingness), leverage (potential to influence the linear model) and influence analysis (represented by the size to point) are represented through influence plots. Data points perturbing the model were identified by high leverage and studentized residuals. Outliers representing RBPs with higher number of changed client mRNAs were identified through high absolute values of standardized residuals. The same was done by (C) plotting number of upregulated clients against number of changed clients, as well as using vector information on (D) differential promoter usage, (E) differential splicing, and (F) differential CDS. (TIF) [file pone.0125745.s001.tif]

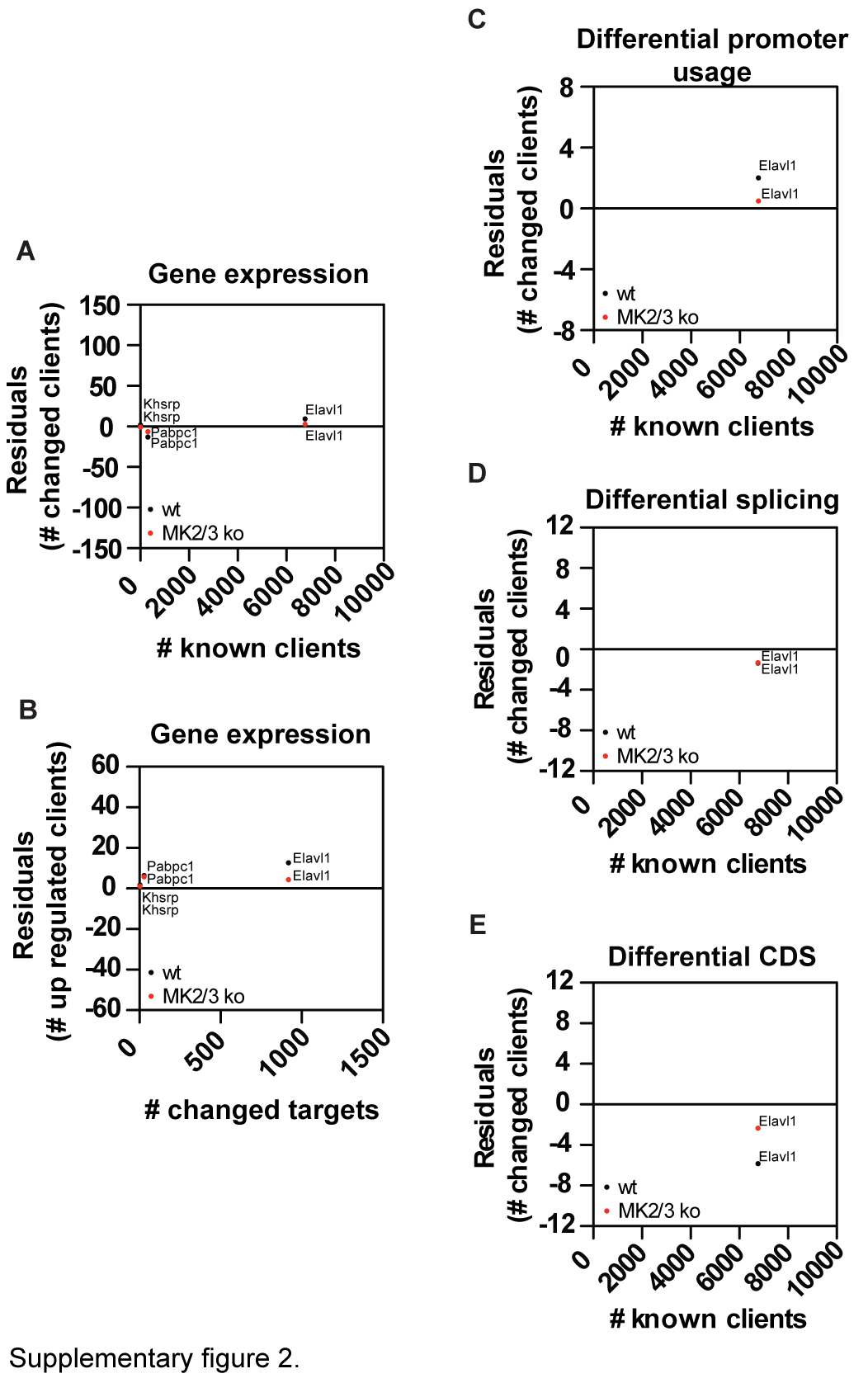

Supplement: S2 Fig — Shown are the RBPs with the highest changes in RNA-protein interactome changes and for which enough information on client mRNAs changes was available. (TIF) [file pone.0125745.s002.tif]

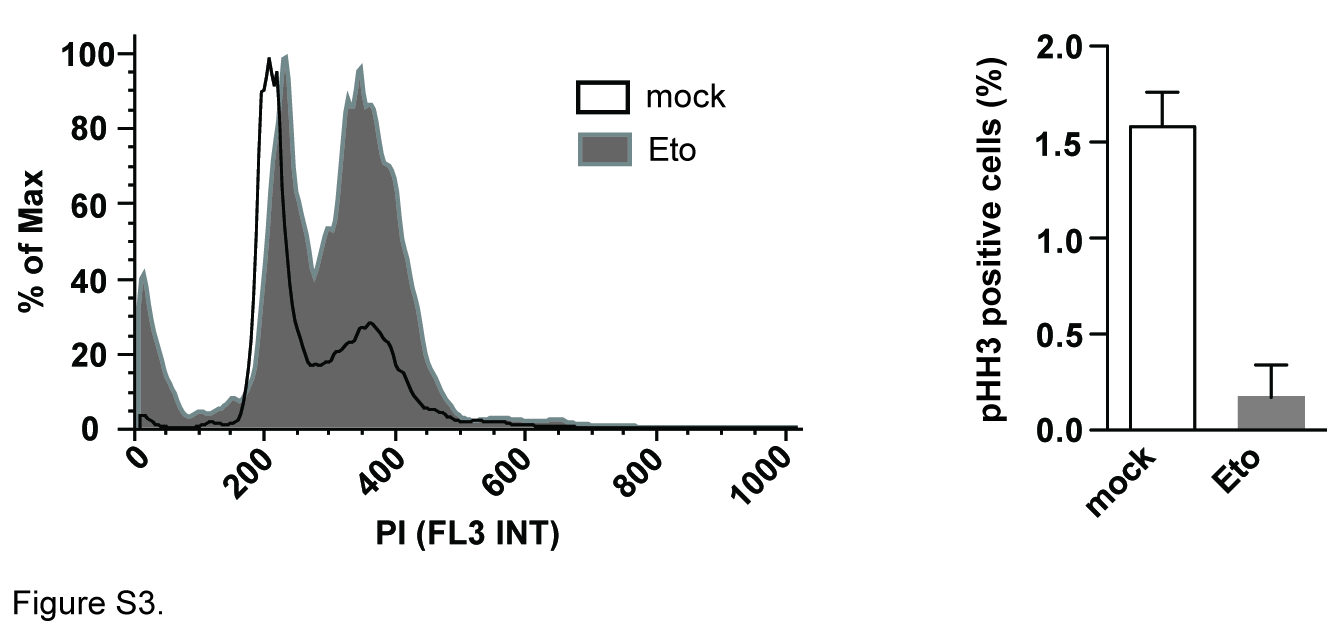

Supplement: S3 Fig — Upon etoposide treatment, Khsrp-/- MEFs arrest in G2 as seen by the increase in cells with a 4N DNA content and decrease in pHH3 positive cells. (TIF) [file pone.0125745.s003.tif]

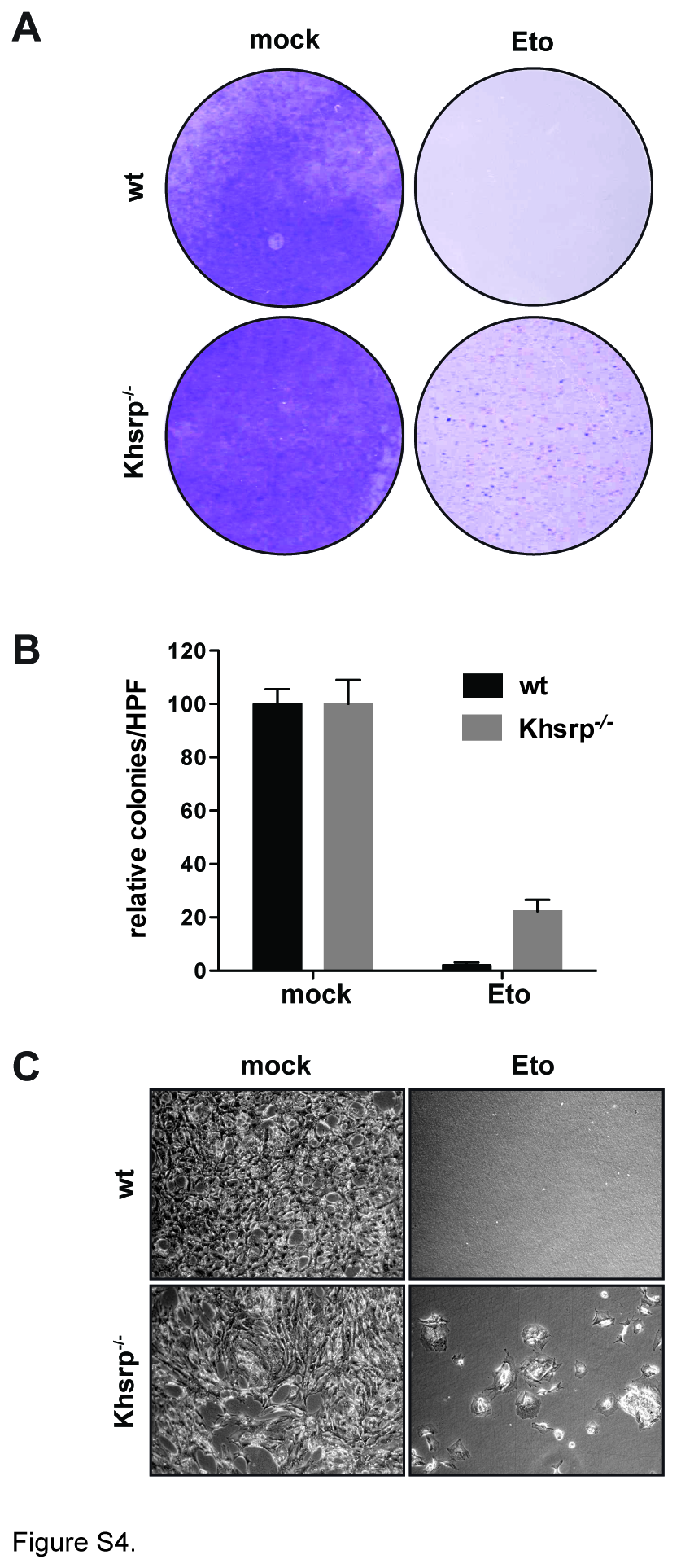

Supplement: S4 Fig — Colony formation assay of etoposide-treated wt and Khsrp -/- cells reveals a significant resistance of Khsrp -/- cells to etoposide treatment. (A) Colony formation assays following a 12 hr exposure to 20μM etoposide are shown for wt and Khsrp -/- cells. (B) Quantification of the data shown in (A). At least 6 high power fields were evaluated for this analysis. (C) Representative microscopic view of the cells shown in (A, B). HPF, high power field; Eto, etoposide. (TIF) [file pone.0125745.s004.tif]
